# Experiences of urine collection devices during suspected urinary tract infections: a qualitative study in primary care

**DOI:** 10.3399/BJGP.2022.0491

**Published:** 2023-05-03

**Authors:** Margaret Glogowska, Caroline Croxson, Christopher Butler, Gail Hayward

**Affiliations:** Nuffield Department of Primary Care Health Sciences, University of Oxford, Oxford.; Nuffield Department of Primary Care Health Sciences, University of Oxford, Oxford.; Nuffield Department of Primary Care Health Sciences, University of Oxford, Oxford.; Nuffield Department of Primary Care Health Sciences, University of Oxford, Oxford.

**Keywords:** general practice, qualitative research, urinary tract infections, urine, midstream, urine collection device

## Abstract

**Background:**

Up to 30% of urine samples from women with suspected urinary tract infection (UTI) are contaminated and need to be repeated, burdening health services and delaying antibiotic prescription. To prevent contamination, midstream urine (MSU) sampling, which can be difficult to achieve, is recommended. Urine collection devices (UCDs) that automatically capture MSU have been proposed as a solution. There are few studies exploring women’s experiences of using such devices.

**Aim:**

To explore women’s experiences of urine collection and the use of UCDs during a suspected UTI.

**Design and setting:**

An embedded qualitative study in a UK randomised controlled trial (RCT) of UCDs among women attending primary care for UTI symptoms.

**Method:**

Semi-structured telephone interviews with 29 women who had participated in the RCT were conducted. The transcribed interviews were then thematically analysed.

**Results:**

Most of the women were dissatisfied with how they normally produced urine samples. Many were able to use the devices, found them hygienic, and would use them again, even if they had initially experienced problems. Women who had not used the devices expressed interest in trying them. Potential barriers to UCD use included positioning for the sample, UTI symptoms making urine collection difficult, and waste disposal because of the single-use plastic in the UCDs.

**Conclusion:**

Most women agreed there was a need for a user- and environmentally-friendly device to improve urine collection. Although using UCDs can be difficult for women experiencing UTI symptoms, they may be appropriate for asymptomatic sampling in other clinical populations.

## INTRODUCTION

Among the bacterial infections managed in primary care, urinary tract infection (UTI) is one of the most common and accounts for 1%–3% of GP consultations.^1^ UTIs occur more frequently in women. Their lifetime risk is 50%, and incidence is estimated at >10% annually.^1^ Asking patients with suspected UTIs to produce urine samples for testing is a common feature of UTI management in primary care, especially during pregnancy or in people with recurrent UTIs.^2^^,^^3^ However, urine samples can be contaminated by the patient’s skin flora or vaginal secretions, producing a mixed growth or equivocal result. This happens in up to 30% of samples from women with suspected UTIs^4^ and repeat testing is often necessary. This places a time and cost burden on health services and affects the women themselves by delaying prescription of an appropriate antibiotic.

To avoid contamination, patients are often advised to discard the first portion of the urine stream, in which the majority of any contaminants may be present, and to collect the midstream portion for the sample;^5^^,^^6^ however, healthcare professionals (HCPs) can often find this process challenging to explain and patients can find it difficult to achieve, especially at a time when urination can be painful, when urine flow can be hard to control, and when quantities of urine produced can be very small.

A proposed solution to the problem of contamination is the use of a urine collection device (UCD) that is designed to automatically capture the midstream portion of a urine stream. There have been three previous studies of these devices,^7^^–^^9^ only one of which — the Whiz Midstream in asymptomatic pregnant women — showed a small benefit in terms of the proportion of contaminated samples.^7^ The authors of the current study recently conducted a three-arm randomised controlled trial (RCT) comparing outcomes from the use of two UCDs (Whiz Midstream and Peezy) to outcomes from standardised practice (which consisted of verbal instructions for collecting a midstream urine [MSU] sample in a sample container — without a UCD — as follows: ‘Please pass the first portion of your urine into the toilet and collect the next portion in this sample pot’), reported elsewhere.^10^

The Whiz Midstream device employs a pressure valve system, designed to let the initial urine stream flow into the toilet and the midstream sample to then flow into a sample bottle.^11^ The Peezy device collects the early urine stream in a sponge, allowing the midstream part to be collected through back pressure.^12^ Illustrations and further details of the UCDs can be found online.^11^^,^^12^

A qualitative study was embedded in the current authors’ RCT^10^ and, using semi- structured interviews, the perceptions and experiences of using the UCDs were explored in a subsample of the women with suspected UTIs who had enrolled in the RCT. The aim was to gather information on the usability and acceptability of the two devices to the women, compared with their usual practice in collecting urine, and this is the focus of the current article. Wider issues around urine collection, such as the women’s awareness of why midstream samples are required and how samples can become contaminated, are presented elsewhere.^13^

**Table 1:** How this fits in

To reduce the problem of contamination in urine samples collected from women with a suspected urinary tract infection, urine collection devices (UCDs) that automatically capture midstream urine have been proposed as a solution. There is, however, very little research exploring women’s experiences of using them. This qualitative study aimed to explore the usability and acceptability of UCDs among women, compared with their usual practice in collecting urine. Many were able to use the devices and found them acceptable, although UCDs may be more appropriate for asymptomatic sampling in other clinical populations.

## METHOD

### Design

Investigation of these issues using qualitative research methods was chosen given the lack of relevant literature and the suitability of qualitative research for investigating individuals’ experiences and perceptions of actions and processes. Semi- structured individual interviews enable researchers to explore topics of interest to them but also to enable participants to spontaneously raise issues they consider important.

### Setting and participants

The RCT recruited adult women (aged 20 years–88 years) presenting to UK general practice with symptoms of UTI, experiencing at least one of: pain on urination; blood in the urine; or frequency of urination; and who were able to give informed consent to take part in the RCT. All RCT participants were asked if they were willing to be contacted about participation in an interview study. In those who agreed to be interviewed, purposive sampling was used to ensure there was a range of ages within each arm of the RCT, and across a range of the GP practices where recruitment was taking place. The authors chose to interview a small number of women who had been randomised to standardised practice to learn about their experiences of producing urine samples and whether they might use a UCD in the future. Women were given written information about the interview study and the opportunity to ask questions.

The women who gave consent were interviewed by telephone as soon as possible after their participation in the RCT had finished, ensuring that their experiences of urine collection were recent. The informed consent process was recorded.

### Data collection

An interview topic guide was developed, taking into account the available literature, which covered issues of interest relating to the UCDs and the women’s wider experiences of providing urine samples at times of suspected UTIs, such as their usual methods of collecting urine, their awareness of the need for MSU sampling, and their awareness of how samples could be contaminated. The topic guide was modified as the study progressed (see Supplementary Information S1). Two experienced female qualitative researchers (a health services researcher and a social sciences researcher, the first two authors) carried out the interviews between December 2016 and March 2018, across the time period when women were being recruited to the RCT. They regularly discussed the interviews to ensure consistency. Data collection finished when the researchers were satisfied that no new issues were emerging from the interviews and agreed that there was the necessary understanding of the emerging categories and themes. The average length of the interviews was 30 min.

### Analysis

The interviews were audiorecorded, transcribed verbatim by a transcription company, and the transcripts checked and anonymised. The data was analysed thematically using NVivo (version 11) to retrieve and organise the data. The researchers read and re-read the transcripts, undertook systematic coding of the data, and established a coding framework. They explored the relationships between codes, leading to the development of categories (provisional groups of codes) and eventually themes, sharing and discussing these with the wider research team to ensure their trustworthiness.^14^ A constant comparison strategy was used in the analysis process,^15^ enabling the researchers to move between different parts of the dataset to check if ideas or categories developed in one part of the dataset were present elsewhere, and ensure that all of the data were comprehensively included. Feedback on the findings was not sought from the participants but the findings were discussed within the research team and with the patient and public involvement contributors.

## RESULTS

In total, 29 women participated in interviews; 13 from the Peezy arm, 13 from the Whiz Midstream arm, and three from the standardised practice arm. Three women declined to take part in the interview study when they were contacted after their participation in the trial ended. The focus was on recruitment among women who had used the devices but a small number of women were also interviewed who were randomised to standardised practice to explore their perceptions of urine sample collection, as well as UCDs and their potential place in UTI care. Participants were aged between 20 years and 88 years. Summaries of their experiences are presented in Supplementary Table S1.

The results of the interviews are presented under the following main themes:

- Initial impressions of UCDs.
- Experiences of using a UCD:
  — learning to use the devices;
  — ease of use; and
  — difficulties experienced.
- Acceptability of UCDs:
  — hygiene;
  — environmental concerns; and
  — cost.
- Willingness to use a device in the future.

### Initial impressions of UCDs

When each participant was asked about how they normally produced urine samples at times of suspected UTIs, many described the difficulties they experienced. They found the small containers usually provided for the purpose hard to use:

*‘They are not very easy to use, a bit messy, a bit inconvenient … not really great.’*

(Participant [P]29, Peezy, aged 35–39 years)

Because of the challenges involved in typically using 30 ml containers in the GP practice toilet to provide a sample, women often chose to keep containers at home for this purpose:

*‘I just think it’s easier at home, you can wash your hands better, it’s just there’s no pressure. I think when you’re in the surgery you feel slightly under pressure.‘*

(P18, Peezy, aged 45–49 years)

To make things easier, some of the women also reported urinating into a larger container and decanting the urine into the smaller specimen pot:

*‘I try and take a sample with me, which I usually have to collect in a plastic jug that I keep specially for the occasion because I find any other little thing is such a job to get it in … you need a bigger thing to collect it. I make sure it’s lovely and clean.‘*

(P9, Peezy, aged 85–89 years)

One participant had found a plastic container that she found very helpful:

*‘I’ve got a plastic oval … It’s rather long but rounded at the ends and that’s fine, it’s plastic and it works beautifully … it looks like a gravy boat.‘*

(P4, no device used, aged 75–79 years)

Because of experiences like these, participants were positive about taking part in the trial of UCDs:

*‘Those little specimen ones can be sometimes quite difficult, so if it’s new it’s got to be a better idea, or I hope it is … I would much rather have a different way of doing it than in one of those pots.‘*

(P3, no device used, aged 70–74 years)

*‘I find it’s always difficult to collect urine … and it’s not very clean the other way as well, so yeah, I think it’s really exciting, someone’s thought about it.‘*

(P15, Whiz Midstream, aged 40–44 years)

### Experiences of using a UCD

#### Learning to use the devices

Participants were given the device, to which they had been randomised, in a bag with instructions printed on it. Women varied in how easy they found it to understand the instructions, especially with the Peezy device:

*‘It’s just presented with these pieces to fit together, it’s like a puzzle in an exam.‘*

(P12, Peezy, aged 70–74 years)

Some participants commented that they had urinary urgency, which limited the time they had to learn how to use the device:

*‘I think it was me trying to be in a rush probably made it harder than it actually was, I think it was very straightforward.‘*

(P14, Whiz Midstream, aged 20–24 years)

However, for most of the participants, learning how to assemble and use their device was manageable:

*‘It was pretty straightforward once you sort of read it and you looked at the pictures to see how it was supposed to fit and things.‘*

(P23, Peezy, aged 55–59 years)

*‘It was quite well explained and there was a diagram on the packet … it was trivial to do … it was easy.‘*

(P19, Whiz Midstream, aged 40–44 years)

Some participants received additional verbal advice from the HCP present:

*‘She explained how to, how it was to do it and everything.‘*

(P2, Peezy, aged 65–69 years)

*‘The practice nurse showed me how to use it, which seemed OK.‘*

(P6, Whiz Midstream, aged 70–74 years)

One participant asked for verbal instructions because she did not have her glasses and was unable to read the written instructions; in most cases verbal advice was offered without being requested.

A number of women expressed surprise at the appearance of the devices, commenting on their unfamiliarity, but they felt that they would become much easier to use with practice:

*‘I think some people could feel quite anxious about it, it’s a very different kind of new thing, new design, but I think women once they get used to it they’ll find it much easier than the old method.‘*

(P15, Whiz Midstream, aged 40–44 years)

#### Ease of use

For both devices, around half of the women, of varying ages, had no difficulties using them in their GP surgery or health centre, finding the process straightforward:

*‘Sometimes you end up with pee all over your hand and miss the pot entirely, but with this one, because it’s like a funnel, it literally, all your wee went straight into it so you didn’t have to worry about missing it.‘*

(P8, Whiz Midstream, aged 25–29 years)

*‘I thought the device was very easy to use and I think it took the stress out of the situation … am I going to go in the pot or am I going to miss it? So I thought it was fine. I thought it was really good. I liked it.‘*

(P18, Peezy, aged 45–49 years)

#### Difficulties experienced

There were, however, others who experienced difficulties in using the devices. Some difficulties were applicable to both devices, in particular those related to collecting urine while having a UTI. Insufficient amounts of urine, lack of control over the urine stream, and pain made urine collection problematic. These difficulties were more commonly described by women from older age groups:

*‘When you have a urine infection, sort of wee comes out when you don’t expect it and it’s very hard to sort of regulate when you can think, well I’ll just go now.‘*

(P9, Peezy, aged 85–89 years)

*‘When you’ve got a water infection you can’t do a lot of urine so … you’ve got to catch it straightaway because you could just do a little drop and that’s the lot.‘*

(P2, Peezy, aged 65–69 years)

The initial urine stream was discarded by the devices. Owing to problems with quantity, this sometimes meant that no, or very little, midstream sample was collected. This was frustrating for participants:

*‘There was virtually absolutely nothing, I mean there was nothing in the device at all apart from what could have been a dribble and by a dribble I mean a minute, the most minute amount and I thought to myself, well this is useless, I can’t give them this sample, this is not a sample, this is just a nightmare!‘*

(P1, Whiz Midstream, aged 65–69 years)

For both devices, some women struggled with not being able to see what they were doing. Some, notably participants aged >65 years, found positioning of the devices difficult:

*‘I tried to sort of peer down and see where it should be and things but it just, it was totally alien to me, I just really thought, oh no … this is not on … I would have had to straddle over an open toilet, which is not the way I normally sit on a toilet.‘*

(P1, Whiz Midstream, aged 65–69 years)

*‘When people have got a water infection you can’t hang about … you’ve got to go then and there … but with that* [the device] *you’re trying to get it in the position … where it’s got to go and then … some of it’s sort of gone before you’ve got it in the position.‘*

(P2, Peezy, aged 65–69 years)

Some participants thought that difficulties with positioning the device might not occur as they became more familiar with using it:

*‘It didn’t catch so much sample as I was expecting it so I had to adjust it on the go … It* [urine] *was dripping all over … Maybe it was just the first time when I didn’t know what to expect and I didn’t know how it really works but maybe second time I would manage it quite alright.‘*

(P21, Peezy, aged 70–74 years)

Because of their difficulties, participants felt that both devices might be unsuitable for less physically fit women, older women, and women with visual impairments:

*‘I was thinking like maybe it could be really difficult for somebody not that physically fit, like for example overweight or elderly people who might struggle to actually reach or put yourself, themselves into the correct position sitting on the toilet.‘*

(P21, Peezy, aged 70–74 years)

*‘I’ve got a friend who has a lot of urine infections … she’s almost blind … she’s got very very poor sight … she would have a great difficulty with one of those things.‘*

(P9, Peezy, aged 85–89 years)

At the same time, participants perceived that the devices could be particularly beneficial in some cases, such as in pregnancy or in people with some movement disorders:

*‘If you don’t have the mobility to make sure you get it in the hole, having a funnel just gives you that little bit more extra sort of leeway, you haven’t got to be so precise, because obviously … people who may have like tremors can’t hold the pot … I think that would be easier for pregnant people because obviously they’ve got that gigantic bump … they may find it difficult to use a regular pot, whereas the funnel kind of, it’s just a lot easier to use.‘*

(P8, Whiz Midstream, aged 25–29 years)

### Acceptability of UCDs

Participants also expressed more general views about the acceptability of UCDs. These were related to hygiene, disposal, and cost.

#### Hygiene

Although urine leakage occurred for some women, some participants felt the devices were more hygienic than the usual method of collecting urine in a 30 ml container:

*‘Definitely cleaner because with the pots like you’d always have to like really scrub your hands afterwards and the pot itself, I know it’s a bit gross, but sometimes the pot itself can get urine on the outside because it’s a bit more difficult. Whereas with this* [device] *like I know that I didn’t have to like clean the pot or anything like that on the outside.‘*

(P17, Whiz Midstream, aged 20–24 years)

#### Environmental concerns

A number of participants expressed concern about the single-use nature of the UCDs:

*‘Because it’s sort of a one-use thing, it seemed a very complex device just to use once and then chuck away, I think, obviously I know you can’t sterilise them again, you can’t reuse them on other people, but from that point of view I’d much rather just wee in a pot like normal.‘*

(P14, Whiz Midstream, aged 20–24 years)

Women commonly raised concerns about disposal of the devices. These were related to their size:

*‘It is obviously a lot bigger than just a normal pot, so it would, if you had every patient using it, the bin would fill up quite easily, quite fast.‘*

(P8, Whiz Midstream, aged 25–29 years)

And the plastic material used:

*‘Well I suppose these days you do think “mm, you know, that is quite a lot of plastic just being thrown away”. I guess if they can design something that’s more environmentally friendly, brilliant … I’m not sure it’s something you put in your recycling really is it?‘*

(P18, Peezy, aged 45–49 years)

Another participant with environmental concerns, who was otherwise in favour of the UCDs, questioned whether there was a possibility they could be manufactured from a material other than plastic:

*‘There was just one thing that troubled me because in our house at the moment we’re looking very carefully at our use of plastics … when they’re discarded you don’t know what’s going to happen to that plastic, where it’s going to end up … and so I realise that probably plastic’s going to be the best material for this particular job, but I just wonder if there are any other materials that could be used … ‘*

(P29, Peezy, aged 35–39 years)

Most of the participants had histories of UTIs and were familiar with providing a urine sample. Many were accustomed to collecting a urine sample at home before attending the GP practice, because it was more convenient and comfortable. For that reason, they questioned whether the devices would be made available in advance, as is the case for the 30 ml containers, and whether they could be reused:

*‘To buy it and keep it at home it’s actually how do you keep it sort of sterilised … would you have to buy one every time or is there a way that it can be sterilised and you can reuse it, that’s the key factor.‘*

(P23, Peezy, aged 55–59 years)

#### Cost

A small number of women commented negatively on the cost of the devices. Although this participant could see a role for the UCDs, especially for those who found using the usual containers difficult, she was concerned that they were more costly:

*‘I gather they’re much more expensive.‘*

(P10, Peezy, aged 80–84 years)

Although the small containers typically supplied by the GP surgeries were often perceived by the women as difficult to use, it was acknowledged that they were not as expensive as new devices were likely to be:

*‘I should imagine that it’s whatever way it’s done it’s going to be a lot more expensive with the new ones than it probably is with the little pots … The little pots that you go to and that we have now, the little specimen jars, is probably a lot cheaper than the new versions that are coming in. ‘*

(P3, no device used, aged 70–74 years)

On the other hand, other participants acknowledged that if UCDs could produce better samples, they could improve the testing process:

*‘… if, you know, it makes the process more accurate, quicker, etc, it’s good.‘*

(P5, no device used, aged 70–74 years)

A few women highlighted that UCDs might also be able to make sure that the right result was achieved first time. In turn, they saw this as a way of reducing need to repeat the urine test and might therefore be cost-effective:

*‘If you can get it right the first time it’s cutting down on so many things, cost involved, the workforce involved.‘*

(P11, Whiz Midstream, aged 55–59 years)

*‘Obviously it’s going to save, people having to look at things that aren’t, you know, it costs money doesn’t it to have to do extra investigations.‘*

(P27, Peezy, aged 25–29 years)

### Willingness to use a device in the future

For both devices, a small number of women commented that they would not be willing to try them again. This was mainly because of a bad experience using the device; but one woman felt them to be unnecessary:

*‘It seemed an elaborate piece of equipment for something that is otherwise very simple and unnecessarily, unnecessary to produce those just for urine samples I thought.‘*

(P10, Peezy, aged 80–84 years)

Many more women, including two of those randomised to standardised practice, said that they would be happy to use a device in the future. Even some who had difficulties using the device the first time would be willing to try again:

*‘I would give it another go, but you know, I’d have to perhaps read it a bit more clearly, and try a bit harder, but I just don’t know if I could, I still don’t know if I’d be able to do it.‘*

(P22, Peezy, aged 70–74 years)

Their reasons included finding a device easier and cleaner to use than their usual method.

Some suggestions were made for how the process could be improved, including clearer instructions and reassurance, changes to the design, and additional equipment:

*‘Even like someone show me how to put it together, like just have one in the office that you could show people you know, how it should fit.‘*

(P15, Whiz Midstream, aged 40–44 years)

*‘If it was a little bit easier to use to get it into the tube … perhaps the design of the thing could be better.‘*

(P22, Peezy, aged 70–74 years)

*‘Maybe there’s a solution where they could have like a frame that could fit on the loo so you’re suspended a little bit and you could just sit into that frame, almost like a chair without a seat.‘*

(P11, Whiz Midstream, aged 55–59 years)

## DISCUSSION

### Summary

For many of the women, their motivation for trying out the UCDs was rooted in their dissatisfaction with how they normally produced urine samples at times of a suspected UTI. The study found that most of the women were not aware of the need for MSU samples and the problem of contamination of the samples until these were mentioned to them as part of enrolment in the trial. Instead, they were eager to take part in the trial as a way of helping to find an easier, ‘less messy’ way of providing a urine sample. When they tried the devices, a large proportion of the women, across all age groups, were able to use them to produce a urine sample in the GP surgery or health centre and would feel happy to use the same device on further occasions, even when they had experienced problems on initial use. Women who had not used the devices in the trial reported that they would like to try them in the future. Thus, both devices were considered acceptable for use at times when the women were presenting to general practice with UTI symptoms and more hygienic than their usual method. The women also suggested that certain groups, for example, pregnant women and those with movement disorders, might particularly benefit from using them.

The use of the UCDs in the trial was facilitated by verbal instructions and support received by the women from the HCP present. Although this helped them to use the devices, it also raises questions about the sustainability of their use in busy and pressured clinical settings, and therefore the feasibility of their introduction into these environments. An important barrier to the use of UCDs relates to the nature of the UTI symptoms themselves and issues around collecting midstream samples, such as producing sufficient quantities of urine and pain on urination. Finding a suitable position in which to collect urine via the UCD was also problematic for some women, especially those who were less mobile. The women also felt that the UCDs would be less usable among women with reduced vision, women who are overweight, or among women with confusion.

The interviews highlighted that it had become common practice for some of the women to collect their urine sample at home before attending an appointment at their GP surgery. Women who experienced recurrent UTIs were often provided with the containers to keep at home ready for use when they suspected a UTI. However, there was uncertainty about whether the UCDs could be made available for home use. Thus, how UCDs could be incorporated into patients’ usual involvement in management of UTIs in the community would need to be carefully considered.

An unanticipated concern that represents a major barrier to use are the issues of the quantity of single-use plastic in the devices and that of waste disposal. These issues, together with the cost of the devices, were considered prohibitive by some women. Among women who experienced recurrent UTIs, they commonly found other solutions to improve their ability to collect samples, such as storing sample pots at home to collect a sample before coming to the GP and using receptacles that worked better for them before transferring their sample to a pot.

### Strengths and limitations

A strength of this study is the range of participants interviewed, particularly across the age groups. The authors found qualitative interviews a very useful method for collecting data about a highly personal issue such as UTIs, and the women felt able to speak with candour and in detail about their experiences.

The women interviewed in this study had participated in an RCT because they were positive about trying out a UCD. This might not be the case among a wider general practice population.

### Comparison with existing literature

Although some of the participants in the current study encountered difficulties in using the UCDs because of UTI symptoms, they suggested that the devices might be particularly suitable for pregnant women. In one other study, a UCD (the Whiz Midstream) was compared with conventional urine collection methods in women attending antenatal clinics. The device was found to be acceptable among these participants, who were not experiencing UTI symptoms.^7^ To the best of the authors’ knowledge, no other studies of UCDs have reported on their acceptability to users.

### Implications for practice

In this study most women confirmed there was an unmet need for a device to improve urine sample collection. The struggles described by these participants when using smaller containers that are usually used in existing practice suggest that more user-and environmentally-friendly collection devices are needed. Some women found that the UCDs tested met that need, and considered them hygienic and easy to use. Others, however, raised concerns about difficulty in using the devices, which in many cases arose because of the symptoms of UTI they were experiencing. Those concerns, however, may not be applicable to asymptomatic sampling in other clinical populations. For this reason, devices may have a role to play when collection of urine is for asymptomatic screening or other clinical purposes.
